# Comparative Analysis of Volatile Profiles from *Rosa gallica* L. Flowers Using HS and SPME Extraction Coupled with GC-MS

**DOI:** 10.3390/foods15142425

**Published:** 2026-07-08

**Authors:** Xiangyang Guo

**Affiliations:** College of Tea and Food Science, Xinyang Normal University, Xinyang 464000, China; xiangyang.guo@ahau.edu.cn

**Keywords:** Gallica rosa (*Rosa gallica* L.), volatile profiles, relative odor activity value (rOAV), principal component analysis (PCA), headspace extraction, solid-phase microextraction

## Abstract

Volatile composition is a core trait that determines the ornamental and application value of *Rosa* flowers. In the current study, the volatile profiles of freeze-dried *Rosa gallica* flowers were comprehensively characterized via gas chromatography-mass spectrometry (GC-MS), coupled with heatmap clustering, principal component analysis (PCA), and relative odor activity value (rOAV) determination. A total of 181 volatile compounds were identified, and distinct volatile profiles were observed between flower samples treated with headspace extraction (RG-HS) and solid-phase microextraction (RG-SPME). Alcohols were the dominant volatile class in RG-SPME, whereas aldehydes were abundant in the RG-HS group. In terms of aroma characteristics, RG-SPME presented stronger green, floral, citrus, and fatty odors, while RG-HS exhibited a more pronounced fruity note. Moreover, sulfurous and dairy notes were unique to RG-SPME and RG-HS, respectively. Additionally, 27 volatiles showed rOAV above one, with 2,3-butanedione and *trans*-citral having the greatest rOAV value of 100, were identified as the potential aroma-active contributors to butter-like and citrus odors in RG-HS and RG-SPME, respectively. These results provide a methodological reference for volatile profiling of *R. gallica* flowers, and support the selection of appropriate extraction methods for future research and resource utilization.

## 1. Introduction

Roses (*Rosa* spp.) are the most important ornamental crops around the world, including 200 species and more than 18,000 cultivars [1]. In addition to the diverse ornamental uses, such as garden, pot plants, and cut flowers, roses are also a source of natural flavorings, fragrances, and pigments, which are broadly applied for the preparation of essential oils, concretes, absolutes, extracts, rose water, or scented teas [2,3], applicating in cosmetics, perfumes, household products, aromatherapy, and foods [4]. In traditional Chinese medicine, roses have been used as a medicament or herbal remedy for the treatment of various diseases, including flu, cold, inflammation, rheumatoid arthritis, osteoarthritis, and chronic pain [5]. Furthermore, roses contain natural bioactive ingredients [6,7], flavonol glycosides, unsaturated fatty acids, carotenoids, procyanidins, phlobatannins, triterpenoids, phenolic acids, polysaccharides, etc., allowing for the excellent nutritional profiles, pharmacological properties, and health benefits, such as antioxidant activity [3], regulating intestinal barrier function [8], neuroprotective activity [9], antimicrobial activity [7], reducing the signs of skin aging [10], anti-inflammatory and analgesic effects [5,11].

Notably, volatile constituents also might be the material basis for biological effects of roses [4,12], which is the reason why rose resources can potentially be developed as an insect attractant [13,14]. Moreover, more than 400 volatile compounds have been identified in previous studies from numerous rose varieties; most of these aroma constituents are derived from phenylpropanoid/benzenoid, terpenoid, and fatty acid pathways [15,16,17]. Although the amounts of volatile compounds account for 0.01–0.03% of the total dry weight of rose flowers, they are crucial for the presentation and enhancement of the overall aroma of roses, influencing the sensory characteristics and economic value of roses [18]. In addition, the volatile constituents also play an important role in signal interaction with plants and stress responses [19]. Flowers are an important organ of roses, with edible and important economic value, as well as wide medicinal properties for the treatment of diseases [20]. Flowers possess rich volatile compounds, which are the raw materials for preparing essential oils or other related products of roses, concrete, rose pure water, absolute, etc. [3]. Moreover, the fragrance of flowers has become an important factor affecting the ornamental value of roses, and is one of the critical factors for attracting consumers. Therefore, it is significant to reveal the characteristics of volatile components and identify the key odorants of rose flowers.

Gallica rosa (*Rosa gallica* L.) from Rosaceae is a common species that is native to Europe and western Asia, and is widely cultivated throughout China. Previous studies on volatile components of *R. gallica* have mainly focused on essential oils and distilled products [7,21]. For example, Shamspur and Mostafavi [22] identified 49 volatile compounds in *R. gallica* essential oil, with citronellol, henicosane, tricosane, and phenylethyl alcohol as the main constituents. A very recent study by Kunc et al. [23] further analyzed the volatile profiles of three native Slovenian rose species, including *R. gallica* and its derived cultivars, using HS-GC-MS, and compared the total volatile content and component categories with *R. damascena*. However, existing studies mostly adopt a single extraction method, and a systematic comparison of different volatile extraction techniques for *R. gallica* flowers is still lacking, which limits the accurate interpretation of volatile data and the selection of analytical methods for different research purposes.

Volatile compounds in flower matrices are characterized by complex composition, low abundance, and a wide range of molecular weights, so the selection of an appropriate pretreatment method is critical for efficient volatile enrichment. Currently, headspace (HS) and solid-phase microextraction (SPME) are commonly used sample pretreatment techniques for enriching volatile compounds from flower matrices. As two solvent-free approaches, HS and SPME follow distinct working principles and exhibit different extraction selectivity. HS captures gas-phase volatiles based on vapor-liquid equilibrium, which is simple to operate and suitable for rapid analysis of highly volatile small-molecule compounds. SPME achieves enrichment through adsorption by the extraction fiber coating, with higher detection sensitivity and wider boiling point coverage, but its results are easily affected by factors such as fiber coating type, extraction temperature, and extraction time. Direct comparison of the two methods on the same *R. gallica* sample can clarify their respective detection biases and applicable scenarios, providing methodological guidance for subsequent studies. As a simple and widely used technique, HS can effectively reduce matrix effects and has been increasingly applied for volatile enrichment in aromatic plants, including flowers [24]. SPME is also an efficient and straightforward approach for volatile compound analysis, suitable for components with low to medium boiling points [25], and has been widely adopted for flower volatile studies [26]. Most importantly, both techniques enable the extraction and enrichment of volatile compounds from complex matrices without organic solvents. Previous studies have shown that the two methods yield distinct compositional profiles in different matrices. For large-leaf yellow tea, SPME predominantly extracts heterocyclic, aromatic, and ester compounds, while HS mainly captures aromatic and heterocyclic compounds [25]. Similarly, in landfill leachates, HS shows better analytical precision, whereas SPME allows faster response without sample dilution [27].

In the present study, the volatile composition and aroma characteristics of *Rosa gallica* flowers were analyzed by gas chromatography-mass spectrometry (GC-MS) technology with HS and SPME aroma extraction, coupled to relative odor activity value (rOAV) determination and principal component analysis (PCA). Results of this study would provide new insights into the potential applications of *Rosa gallica* flowers in cosmetics, pharmaceuticals, food, and non-food products, which is beneficial for the breeding and improvement of rose varieties.

## 2. Materials and Methods

### 2.1. Reagents and Materials

The fresh flowers of Rosa (*Rosa gallica* L.) were harvested early in the morning before sunrise from Zhengzhou City (Henan province, China) at the flowering stage in May 2022. Detailed information on the Rosa samples is shown in the Supplementary Materials. All flower samples were pooled and mixed uniformly to reduce individual differences. The freeze-dried flower samples, which were conducted by use of a freeze dryer (ALPHA 1-4 LD plus, Martin Christ Freeze Dryer, Martin Christ Gefriertrocknungsanlagen GmbH, Osterode am Harz, Germany), were powdered in an ultra-mill (Shanghai Dianjiu Chinese Machinery Manufacturing Co., Ltd., Shanghai, China) to pass through the 350 μm sieve, for the subsequent volatile analysis.

### 2.2. Headspace (HS) Extraction of Volatile Compounds in Flowers

Volatile compounds were obtained by headspace extraction from *Rosa gallica* flowers (RG-HS), which refers to the previous studies described by Guo et al. [25], with minor modifications. Samples of 2.0 g (dry weight) were placed into a 20 mL headspace vial, and then the vial was covered using a headspace auto-loading crimp cap with septa. An Agilent 7697A headspace injector (Agilent Technologies, Inc., Santa Clara, CA, USA) coupled to an Agilent 7890A GC (Santa Clara, CA, USA) was used to analyze the volatile compounds from flowers of *Rosa gallica*. The oven temperature, transfer-line temperature, and manifold temperature were set at 120 °C, 150 °C, and 140 °C, respectively. The injection time was 60 s, and the incubation time was 20 min.

### 2.3. Solid Phase Micro-Extraction (SPME) for the Enrichment of Volatile Compounds in Flowers

The volatile compounds of *Rosa gallica* flowers were extracted using the SPME method (RG-SPME) with slight modifications [25]. Briefly, the *Rosa gallica* flower samples were weighed (2.0 g) and transferred into a 50 mL beaker flask sealed with foil. Subsequently, the prepared flower samples were placed into a 45 °C water bath for equilibration (6 min). A SPME fiber (50/30 μm layer of DVB/CAR/PDMS, Supelco, Inc., Bellefonte, PA, USA) was applied to extract the volatile compounds at 45 °C, while the extract was held for 45 min. And then, the volatile compounds were desorbed (5 min) by loading the SPME fiber into a GC injector in a split ratio of 10:1.

### 2.4. Volatile Analysis by GC-MS Technology

For GC-MS analysis, a GC (Agilent 7890A) coupled with a mass spectrometer (Agilent, Santa Clara, CA, USA) was used, with a fused-silica capillary column (DB-5, 60 m, 0.25 mm ID, and 0.25 μm film thickness, J&W, Folsom, CA, USA) for the separation of volatile components. The GC operating conditions were the same as described by Guo et al. [25], as follows: initial temperature was 40 °C, maintained for 2 min, increased to 190 °C at a rate of 3 °C/min and held for 2 min, subsequently raised at 10 °C/min to 230 °C, maintained for 2 min. The carrier gas was helium (37 cm/s) with a purity of ≥99.999%. The mass spectrometer was operated in the electron-ionization (EI) mode at an ionization voltage of 70 eV. The transfer line was set at 250 °C, and the temperature of the ion source was 250 °C. The mass spectra were acquired in full-scan mode with a range of 30–500 amu. Retention indices (RI) were calculated from the retention time of C5~C28 *n*-alkanes by linear interpolation. The peak was deconvoluted, and the identification of the volatile compounds was based on comparison with the NIST Mass Spectral Library (2017 version), retention index (RI), references, and an in-house database. Additionally, some volatile compounds were positively identified by matching them with standard compounds (Table S1). Blank control experiments were performed for both extraction methods, in which empty vials without a flower sample matrix were processed and injected following identical HS and SPME analytical protocols. All peaks detected in blank runs were subtracted from the sample chromatograms to exclude background interference from the injection system, vial septa, and SPME fiber coating. Only compounds confirmed to be derived from the flower matrix were retained for subsequent volatile profiling and aroma analysis. The relative abundance of each identified volatile compound is calculated via peak area normalization (the ratio of individual peak area to the total peak area of all detected compounds) and expressed as relative peak area (%), and all data are semi-quantitative and do not represent absolute concentrations.

### 2.5. Determination of Relative Odor Activity Value (rOAV)

In this work, the rOAV was calculated as the ratio of the relative peak area of each volatile compound to its literature-reported odor threshold. The rOAV results were used for the preliminary screening of potential aroma-active compounds and for the relative comparison of odor contribution profiles between the two extraction methods [28]. The calculation of rOAV is shown below:

(1) $$rOAV\left(\%\right)=\frac{OAVi}{OAVmax}\times 100=\frac{\frac{C_{i}}{T_{i}}}{\frac{Cmax}{Tmax}\;}\times 100=\frac{\frac{{Ra}_{i}}{T_{i}}}{\frac{Ramax}{Tmax}\;}\times 100$$

where OAVi is the odor activity value (OAV) of a volatile component, which is determined by using the ratio of the concentration (Ci) of each volatile component to its corresponding odor threshold value (Ti) in air. As for the ‘*max*’, it is the volatile component that has the highest OAV value. Rai is the relative amount (%) of the volatile component in *Rosa gallica* flower samples. And the rOAV of the volatile component, which has the OAV*max*, is regarded as 100.

### 2.6. Statistical Analysis

Statistical analysis was performed using SPSS Statistics (version 22.0 for Windows, SPSS Inc., Chicago, IL, USA). An independent-samples *t*-test was used to compare the relative peak area of each volatile compound between the RG-HS and RG-SPME groups, and differences with *p* < 0.05 were considered statistically significant. Given the multiple-testing risk caused by simultaneous testing of multiple compounds, all significance results were interpreted in combination with relative abundance difference and biological significance. All data are presented as mean ± standard deviation.

## 3. Results and Discussion

### 3.1. Volatile Properties of Rosa gallica Flowers

**Figure 1:**
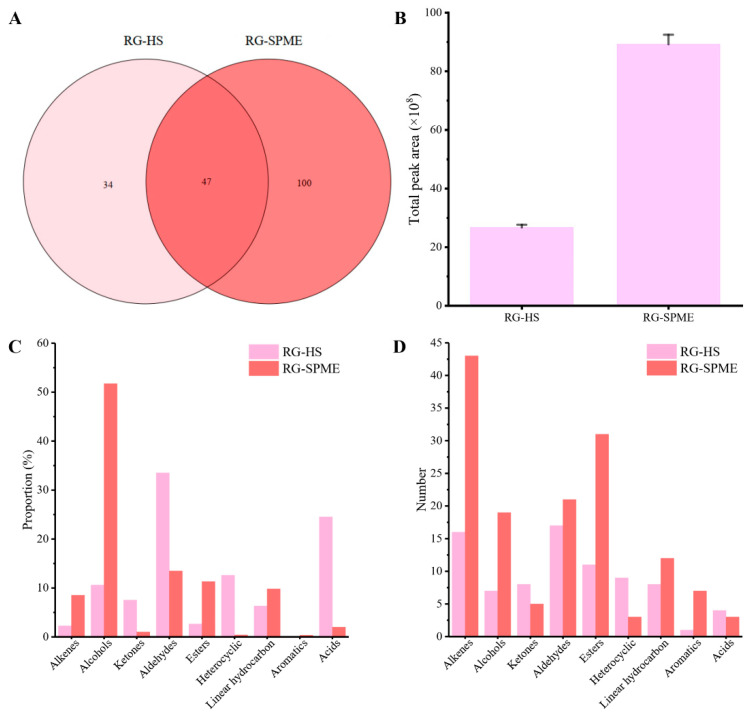
Volatile profiles of *Rosa gallica* flowers extracted by the HS and SPME method. (**A**) The Venn diagram of different volatile compounds in *Rosa gallica* flowers. (**B**) The total amount of volatiles in *Rosa gallica* flowers. The proportion (**C**) and number (**D**) of the volatile categories in the flower aroma from *Rosa gallica*.

In total, 181 volatile compounds were identified in *Rosa gallica* flowers by the two extraction methods combined (Table S2). Eighty-one and 147 volatiles were found in RG-HS and RG-SPME, respectively, and 47 volatile components were shared by the two samples (Figure 1A). Notably, the RG-SPME group detected more identified compounds and higher total relative abundance (Figure 1B). These identified volatiles can be classified into nine groups, including alkenes, alcohols, ketones, aldehydes, esters, heterocyclics, linear hydrocarbons, aromatics, and acid compounds, according to their chemical structure. As displayed in Figure 1C,D, the number and proportion of volatile categories of identified volatiles were very different between RG-HS and RG-SPME. The number of alkenes, aldehydes, and esters was abundant, which was more than 10 in both samples. In addition, 19 alcohols and 12 linear hydrocarbon compounds were found in RG-SPME, while the quantities in RG-HS were 7 and 8, respectively. As for the proportion of identified volatiles, alcohols and aldehyde compounds were dominant, accounting for more than 10.0% in *Rosa gallica* flowers. Furthermore, alcohols and aldehyde compounds were the most abundant in RG-SPME and RG-HS, respectively, representing for 51.73% and 33.50% of the total relative abundance in RG-SPME and RG-HS, respectively (Figure 1C). Ester compounds were the majority in RG-SPME, and heterocyclic compounds accounted for 12.61% of the total relative abundance in RG-HS, which was 31.65 times that in RG-SPME. Although the number of alkene compounds was high, their proportions were relatively small in both samples, accounting for 2.25% and 8.50% in RG-HS and RG-SPME groups, respectively. Representative total ion current chromatograms of the RG-HS and RG-SPME groups are provided as Supplementary Figure S1.

#### 3.1.1. Volatile Profiling of RG-HS

**Figure 2:**
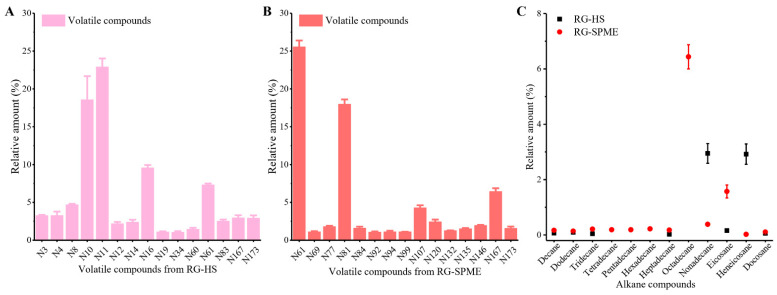
The volatile compounds with relative amounts higher than 1% in RG-HS (**A**) and RG-SPME (**B**), and the linear hydrocarbon compounds in both samples (**C**). N3, 2-methylpropanal; N4, 2,3-butanedione; N8, 2-methylbutanal; N10, pentanal; N11, acetic acid; N12, 1-hydroxy-2-propanone; N14, hexanal; N16, furfural; N19, 2-furanmethanol; N34, hexanoic acid; N60, nonanal; N61, phenylethyl alcohol; N69, octanoic acid; N77, methyl nonanoate; N81, 3,7-dimethyl-2,6-octadien-1-ol; N83, geraniol; N84, *trans*-citral; N92, methyl decanoate; N94, eugenol; N99, *cis*-geranyl acetate; N107, dodecanal; N120, 1-dodecanol; N132, tridecanal; N135, methyl dodecanoate; N146, tetradecanal; N167, nonadecane; N173, heneicosane.

A total of 81 volatiles were observed in RG-HS, among which acetic acid, pentanal, furfural, and phenylethyl alcohol were the major volatiles with the relative abundance higher than 7.0%. The first two compounds comprised 41.46% of the total relative abundance in RG-HS. And acetic acid, having a pungent and vinegar-like flavor [29], is also observed in raw Pu-Erh tea during the steeping process [30]. Pentanal with putative winey, fruity, or berry-like odors is the aroma-active compound in oolong tea [31]. Furfural imparts caramel-like and sweet odors, which are formed with serine as the potential aroma precursor by Strecker degradation during thermal treatment, and also is the main aroma compound in water-distilled oil of flowers from *R. gallica* and *R. kazanlik* [21]. The latter one, exhibiting floral and rose-like odors, is the key aroma contributor of floral note in teas [31,32,33]. The floral flavor compounds, such as nonanal (1.46%) and geraniol (2.52%), were also abundant. Nonanal is commonly found in flower aroma from rose varieties, and is the character aroma compound in *Allium tenuissimum* L. flower with a low level of threshold value [18,26]. Geraniol is considered to be the key aroma contributor to rose-like, sweet, and honey-like odors in teas [31,34], which is the major component in the essential oil of *Amomum tsao-ko*, and might be the potential material basis for the antioxidant activity [35]. The green flavor compound of hexanal with a grassy note is the fatty acid derivative arising from linolenic acid [16], which was identified in RG-HS with a high relative abundance of 2.39%. In addition to the volatiles mentioned above, 2-methylpropanal, 2,3-butanedione, 2-methylbutanal, 1-hydroxy-2-propanone, 2-fruanmethanol, hexanoic acid, nonadecane, and heneicosane also had a relatively high relative abundance above 1.0% (Figure 2A). The latter two linear alkane compounds are also detected in the essential oil of *Rosa gallica* [21]. The former two components imparting malty and butter-like odors, respectively, are the major aroma compounds in oolong tea infusion [31]. 2-Methylbutanal, showing fruity or coffee-like flavor, can be detected in European vinegar [29] and is regarded as the aroma-active compound in Chinese yellow tea based on measured aroma intensity [36]. 2-Fruanmethanol, conveying burnt, sweet, or caramel-like odors, is the main volatile compound in summer green tea [33] and large-leaf yellow tea [37].

Additionally, the fruity 3-methylbutanal, 6-methyl-5-hepten-2-one, (*E*)-3-hexen-1-ol acetate, ethyl octanoate, methyl nonanoate, and ethyl nonanoate had a relatively low relative abundance, which was less than 1.0% in RG-HS. It has been reported that the former two compounds are the key aroma components in the *Allium tenuissimum* L. flower [26]. And 6-methyl-5-hepten-2-one is considered to be the aroma-active compound in green tea due to its high aroma intensity [31], and is also found in flower aroma from different rose varieties [18]. Besides hexanal, the green flavor compound, such as heptanal, and *β*-cadinene with grassy and minty odors were found in relatively low amounts. And the latter one can be detected in the essential oil of *Rosa canina* flower [38]. 2-Methylfuran, 2-acetylfuran, and dihydro-2-methyl-3(2H)-furanone impart a nutty note, might be generated in foods by the Maillard reaction during heating [37,39]. 5-Methyl-2-furancarboxaldehyde and 2-acetylpyrrole are commonly identified in teas with thermal treatment [32,37]. Previous studies reported that 2-acetylpyrrole can be produced from the thermal reaction of L-theanine with D-glucose by heating above 150 °C [40]. *D*-Germacrene, which has a woody or spicy flavor, has been identified as a major compound in mastic [41]. The woody odor compounds α-pinene, camphene, terpinolene, pyranone, and caryophyllene, which exhibit pine-like, camphor-like, hay-like, and woody notes, respectively, are observed as main components in the essential oils of aromatic plants and contribute to their antioxidant activity [42].

Significantly, much more volatile compounds having a floral note, benzaldehyde, *cis*-geranyl acetate, benzeneacetaldehyde, linalool, phenylethyl formate, phenylethyl acetate, eugenol, methyleugenol, etc., were identified in RG-HS with a relatively low content. The former two are widely distributed in flower aroma from different rose varieties [18], and the latter two, imparting a clove-like odor, might be the potential key odorants in piper [43]. Linalool, which might be obtained by hydrolysis of its glycoside precursor during rose flowering [15], promotes the generation of floral and sweet odors in teas [40], and also is the key aroma compound in cinnamon [44]. The remaining four aroma compounds have characteristic rose-like, floral odors and relatively low odor threshold (0.0002~0.27 mg/Kg in air), which appeared to have high odor activity values (OAVs) in teas or vinegar [29,31,32]. In addition, to the best of our knowledge, pineapple ketone with sweet and strawberry-like odors was detected in *Rosa gallica* flowers for the first time under the present analytical conditions. This compound has not been reported in previous volatile profiling studies of *R. gallica*, including the recent work of Kunc et al. [23]. Nevertheless, this preliminary finding still requires further verification across more sample batches and germplasm resources.

#### 3.1.2. Volatile Profiling of RG-SPME

In total, 147 volatile compounds were identified in RG-SPME, among which phenylethyl alcohol showed the highest relative peak area, which was consistent with previous reports on *R. gallica* essential oil and flower volatile profiles [22,45]. It is an important aroma compound giving for strong floral and rose-like scents for aromatic plants, which primarily occurs in its glycosidic form in the unopened flowers of the rose; glycoside hydrolysis is beneficial for its liberation during blooming [15]. 3,7-Dimethyl-2,6-octadien-1-ol, octanoic acid, and 1-dodecanol were only identified in RG-SPME, with a high relative abundance above 1.0%. The latter two imparted rancid and earthy odors, respectively, and the former one contributed to floral, rose-like, or citrus odors with a relatively high abundance of 17.98%, which is the principal bioactive constituent in medicinal plants [46]. Similar to the cases of RG-HS, the linear hydrocarbon compounds, such as nonadecane and heneicosane, were detected in RG-SPME at relatively high abundance of 6.44% and 1.57%, respectively. Notably, the quantity of nonadecane in RG-SPME was significantly greater than that in RG-HS, whereas the amount of heneicosane exhibited the opposite trend, being lower in RG-SPME compared to RG-HS. As for the volatiles, methyl nonanoate, *trans*-citral, methyl decanoate, eugenol, *cis*-geranyl acetate, dodecanal, tridecanal, methyl dodecanoate, and tetradecanal, which were detected in RG-SPME with the relative abundance higher than 1.0% (Figure 2B), were larger in relative abundance than those in RG-HS. Methyl nonanoate, with sweet and pine-like odors, can convey a fruity scent for dry sausages [47]. Methyl decanoate, dodecanal, methyl dodecanoate, and tetradecanal exhibit fatty or waxy flavor, and the former one is also an important contributor of fruity odor in wine [48,49]. The floral note aroma eugenol and *cis*-geranyl acetate, showing clove-like and neroli-like odors, respectively, are widely found in spice or rose varieties [18,43,44]. Tridecanal with waxy or citrus scents can be detected in water-distilled oil from the flowers of *Rosa kazanlik* [22]. *trans*-Citral, which is of lemon-like scent, has been identified as an important aroma component contributing to the citrus odor of rose-related products [14,45].

Acetic acid, 2-furanmethanol, hexanoic acid, pentanal, hexanal, furfural, nonanal, and geraniol, which were abundant in RG-HS, were not detected or were identified at a relatively low level in RG-SPME. However, up to 100 volatiles were solely identified in RG-SPME. These differences are mainly attributed to methodological variations in extraction preferences and analytical performance between HS and SPME [25,27]. Dimethyl sulfide is a key odorant contributing to the fresh flavor of green tea and has been identified as an aroma-active compound in Jingshan green tea [50]. Ethyl acetate and ethyl decanoate have the characteristic fruity odor, and 2-hexenal, (*E*)-2-hexenal, (*E*,*E*)-2,4-hexadienal, (*E*,*E*)-2,4-heptadienal, (*E*,*Z*)-2,6-nonadienal, (*E*)-2-nonenal, as well as methyl salicylate, imparting green note, were uniquely identified in RG-SPME. (*E*)-2-Hexenal and (*E*)-2-nonenal have strong leafy and cucumber-like flavor, respectively, which are the fatty acid-derived volatile organic compounds with linolenic or linoleic acid as the precursors [16]. 2-Hexenal possesses strong grassy and herbal scents for tea [32]. (*E*,*E*)-2,4-Hexadienal, (*E*,*E*)-2,4-heptadienal, and (*E*,*Z*)-2,6-nonadienal, which exhibit green notes or faint cucumber-like and melon-like odors, are reported as the key odorants in the aroma of oolong tea infusion based on the determined aroma intensity [31]. Methyl salicylate, proven to be a contributor to the minty odor, is a product of glycoside hydrolysis in teas [40].

With regard to the characteristic rose flavor, several volatile compounds with rose scent or other floral odors, such as benzyl alcohol, methyl benzoate, indole, *trans*-*β*-ionone, citronellol, acetophenone, *α*-farnesene, nerolidol, *trans*-isoeugenol, and (*E*)-farnesyl acetate, exhibiting pleasant floral and sweet aroma notes, were only identified in RG-SPME. These volatiles, together with linalool, benzaldehyde, and phenylethyl acetate, are the major aroma compounds from Rosa varieties in the previous studies [14,18,22,45], whereas they were present in only small amounts in the flower aroma of *Rosa gallica* of this study. On the other hand, linalool oxides, the main aroma compounds in *Rosa alba* [18], were not detected in *Rosa gallica* flowers.

It is noteworthy that many more linear alkanes were found in both samples (Figure 2C), which is in agreement with the previous studies that linear hydrocarbon compounds were abundant in the aroma compositions of Rosa varieties [18,45]. These alkane compounds themselves have no odor, but they can act as perfume fixatives, thereby facilitating the presentation of the overall aroma of *Rosa gallica* flowers.

### 3.2. Differential Volatile Profiles of Rosa gallica Flowers from Different Extraction Methods

**Figure 3:**
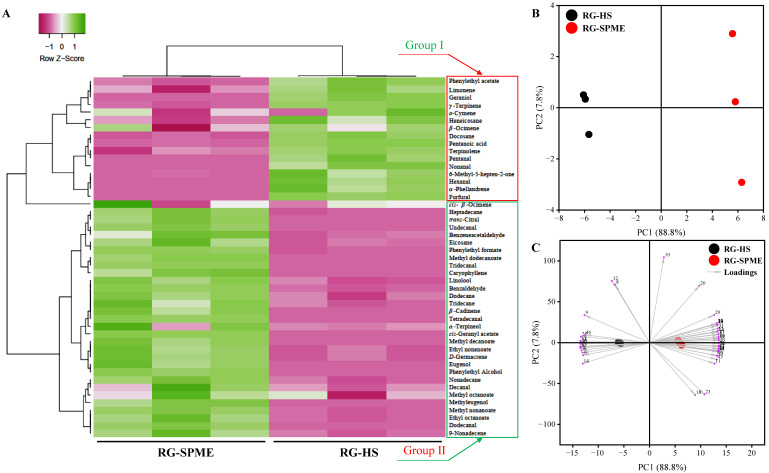
Heatmap and PCA analysis of *Rosa gallica* flowers. (**A**) Heatmap analysis on commonly identified volatiles. (**B**) The score plot of PCA analysis. (**C**) The biplot of PCA analysis. Compound nos. correspond to Table S3.

A heatmap was generated to visualize differences in the volatile profiles of *Rosa gallica* flowers from different extraction methods, based on 47 common volatile compounds (Table S3). As shown in the heatmap (Figure 3A), color coding was graded from purple to green with relative intensity increasing from low to high. The two cuts from *Rosa gallica* flowers were clearly divided into two categories corresponding to HS and SPME extraction. Meanwhile, the 47 common volatile compounds were classified into two groups (Group I and Group II, shown in Figure 3A), of which Group I represented the volatile compounds with higher relative abundance in RG-HS than in RG-SPME, while Group II showed the opposite trend, indicating distinct differences in volatile distribution between the two extraction methods.

To further intuitively perceive the differences in aroma properties between the two samples, PCA analysis was conducted on the 47 common volatile compounds (Table S3). As can be seen from Figure 3B,C, the two flower samples (RG-HS and RG-SPME) were well separated, confirming significant differences in their volatile profiles. Specifically, Figure 3C shows that RG-SPME scored high in the positive direction of PC 1, which was dominated by compounds with high loadings such as phenylethyl formate, *cis*-geranyl acetate, methyl nonanoate, and *trans*-citral, the characteristic aroma compounds with floral, fruity, or citrus scents, which are more effectively captured by the SPME method. In contrast, RG-HS showed high scores in negative PC 1, where the loadings of volatile compounds, including pentanal, *γ*-terpinene, nonanal, geraniol, etc., which were abundant in the HS-extracted samples, reflected the selectivity of the HS method for specific volatile components.

From the methodological perspective, the distinct volatile profiles obtained by HS and SPME are driven by the combined effects of their inherent extraction principles and operating conditions. HS extraction relies on gas–liquid equilibrium to capture low-molecular-weight, highly volatile compounds without direct contact with the sample matrix [27]. It performs better for low-boiling-point, high-vapor-pressure components, but the high incubation temperature (120 °C) used to promote analyte vaporization may also induce thermal degradation of heat-sensitive substances and Maillard-type reaction, resulting in the generation of heat-derived compounds such as furfural and 2,3-butanedione. In contrast, SPME achieves analyte enrichment via fiber adsorption, and the lower extraction temperature (45 °C) better preserves heat-sensitive components, with higher enrichment efficiency for medium- and low-volatility terpenes and alcohols [25]. Accordingly, the profile difference observed in this study reflects the overall discrepancy between the two complete technical schemes, rather than merely the difference in extraction selectivity. Combined with the heatmap and PCA analyses, the significant divergence in the volatile profiles of *R. gallica* flowers obtained by the two methods is further verified at the multivariate statistical level.

Even when adopting the same HS-based analytical strategy, volatile profiling results may still vary considerably across studies due to differences in experimental parameters. Compared with the study of Kunc et al. [23], which also used HS-GC-MS to analyze *R. gallica* flowers, both studies detected phenylethyl alcohol, henicosane, and nonadecane, confirming the stability of these marker compounds. Nevertheless, noticeable differences existed in the total number of identified compounds and the relative proportion of each component category. Alkenes accounted for a higher proportion in the study by Kunc et al. [23], while aldehydes and alcohols were more abundant in our work. This difference is mainly attributed to variations in sample pretreatment (fresh flowers vs. freeze-dried flowers), HS incubation temperature, chromatographic column type, and other analytical parameters, which further demonstrates that extraction and detection conditions exert a significant impact on the volatile profiling results of *R. gallica* flowers. This finding aligns with the conclusion of Kim et al. [14] that extraction conditions markedly shape the volatile composition of rose flowers. These variations provide a theoretical basis for selecting extraction approaches based on research objectives. HS is more suitable for the rapid detection of high-volatility components, while SPME is preferred for comprehensive profiling of overall volatile characteristics.

### 3.3. Odor Profiles of Rosa Gallica Flowers from Different Extraction Methods

**Figure 4:**
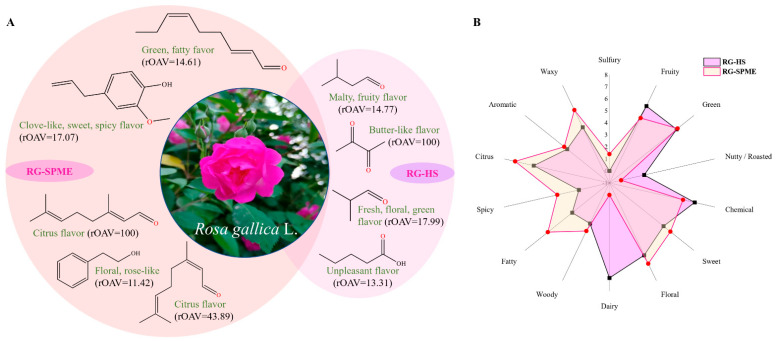
The potential key aroma compounds (**A**) and aroma radar plot (**B**) of *Rosa gallica* flowers. (**A**) The aroma compounds with rOAV above 10 in RG-HS and RG-SPME. (**B**) The aromatic series of volatile compounds in *Rosa gallica* flowers based on rOAVs with logarithmic computations.

The volatile compounds in a certain composition and concentration constitute the complete flower aroma, and the influence of each volatile compound on the overall aroma depends on the content and odor threshold. In this work, the relative odor activity value (rOAV) was applied for evaluating the contribution of volatile compounds to the odor of flowers [28]. In general, the volatile component with rOAV above one is regarded as the aroma-active compound of the sample, while the volatile compound with 0.1 ≤ rOAV < 1 plays an important role in modifying the overall odor characteristics of the samples [51]. A total of 36 aroma compounds were found with the rOAV greater than 0.1 (Table S4), of which 21 volatiles had the rOAV values above one (Table 1). A total of 9 and 16 volatile compounds with an rOAV higher than one were identified in RG-HS and RG-SPME, respectively. Four volatile compounds, including pentanoic acid, nonanal, phenylethyl alcohol, and *trans*-citral, were shared between the two samples, all with an rOAV above one. Based on estimated rOAV values, 2,3-butanedione, which imparts a butter-like odor, had the largest rOAV value (rOAV = 100) in RG-HS, and trans-citral showed the highest rOAV value (rOAV = 100) in RG-SPME. Both compounds were preliminarily identified as potential key aroma-active compounds, and their actual aroma contributions remain to be further verified by GC-olfactometry (GC-O) and aroma recombination experiments in future studies. Moreover, a total of 9 aroma compounds were observed with the rOAV values above 10, which might be the key odorants with great potential in each sample. The 9 volatile compounds were divided into two categories, each representing the aroma compounds with higher rOAV values in one rose flower sample compared to the other one (Figure 4A).

Volatiles exhibiting similar odor qualities can be classified into a group, shaping an aroma spectrum with several odor attributes to establish the aroma profile of the *Rosa gallica* flower [32]. Fourteen odor attributes, fruity, green, floral, citrus, etc., were confirmed based on their odor descriptors (Table S2). And the unpleasant flavor, rancid and solvent-like odors, which were considered to be inappropriate for the rose flower, belonged to the chemical attribute. Accordingly, to further display the differences in odor profiles of *Rosa gallica* flowers from different extraction methods, the image of aroma profiles of volatiles for two samples was plotted, and the coordinates were the sum of rOAVs of the volatiles with the same odor attributes, with logarithmic computation.

Sulfur and dairy attributes were uniquely recorded in RG-SPME and RG-HS, respectively, and the remaining odor types were shared by both samples (Figure 4B). As for the common odor types, the fruity, nutty/roasted, and chemical attributes in RG-HS had higher intensity than those in RG-SPME, while the others showed the opposite trend. The RG-SPME possessed a high intensity of citrus odor, which might be from the contribution of *trans*-citral with the largest rOAV value. And 2,3-butanedione was the greatest potential contributor of dairy note for RG-HS.

## 4. Conclusions

In this study, volatile compound profiles of freeze-dried *R. gallica* flowers obtained by HS and SPME coupled with GC-MS were comparatively analyzed, combined with multivariate statistical analysis and rOAV determination. A total of 181 volatile compounds were identified, among which 47 volatiles were commonly found in two samples. The two extraction methods produced significantly different volatile profiles. RG-SPME detected a larger number of compounds with alcohols as the dominant class (51.73% of total relative abundance), while RG-HS was dominated by aldehyde compounds. Based on rOAV values, 21 potential aroma-active compounds (rOAV > 1) were preliminarily screened. 2,3-Butanedione with butter-like odor and *trans*-citral with citrus-like odor showed the highest relative rOAV in RG-HS and RG-SPME, respectively. Fruity, green, and floral odor attributes were detected in both extraction methods, while the dairy note was unique to RG-HS, and the sulfury note was only found in RG-SPME. This study clarifies the detection bias and applicable scenarios of the two extraction methods, and provides methodological reference and baseline data for future volatile profiling research and the deep processing utilization of *R. gallica* flowers.

## Figures and Tables

**Table 1:** Volatiles with rOAV above one in *Rosa gallica* flowers from different extraction methods.

Volatile Compounds	rOAV	
	RG-HS	RG-SPME
2-Methylpropanal	17.99	0.00
2,3-Butanedione	100.00	0.00
3-Methylbutanal	14.77	0.00
Acetic acid	9.72	0.00
Pentanoic acid	13.31	2.37
Hexanoic acid	1.21	0.00
Nonanal	2.59	1.50
Phenylethyl alcohol	1.92	11.42
(*E*,*Z*)-2,6-Nonadienal	0.00	14.61
(*E*)-2-Nonenal	0.00	7.47
Octanoic acid	0.00	2.03
Decanal	0.71	1.24
*cis*-Citral	0.00	43.89
*trans*-Citral	3.41	100.00
Eugenol	0.71	17.07
Dodecanal	0.06	1.22
Methyl undecanoate	0.00	2.52
*α*-Muurolene	0.00	1.74
Methyl dodecanoate	0.11	5.46
Ethyl dodecanoate	0.00	2.68
Benzyl Benzoate	0.00	2.07

## Data Availability

The original contributions presented in this study are included in the article/Supplementary Materials. Further inquiries can be directed to the corresponding author.

## Supplementary Materials

The following supporting information can be downloaded at: https://www.mdpi.com/article/10.3390/foods15142425/s1, Figure S1. Representative total ion current chromatograms of RG-HS and RG-SPME. Table S1. The related information of chemicals used in the research. Table S2. Odor quality, odor threshold value, and relative amounts of the identified volatiles in *Rosa gallica* flowers from different extraction methods. Table S3. The common volatiles identified in *Rosa gallica* flowers from different extraction methods. Table S4. The rOAV values of identified volatiles in *Rosa gallica* flowers from different extraction methods.
