# Supplementary material for: Comparative Analysis of Volatile Profiles from Rosa gallica L. Flowers Using HS and SPME Extraction Coupled with GC-MS
Source: Foods. 2026 Jul 8;15(14):2425. doi: 10.3390/foods15142425 (PMC13409530; doi:10.3390/foods15142425)
Supplement: Supplementary file 1 [file foods-15-02425-s001.zip › foods-4404567-supplementary.pdf]

Supplementary material

Comparative analysis of volatile profiles from *Rosa gallica* L. flowers using HS and SPME extraction coupled with GC-MS

(Abbreviated running title: Aroma profiles of *Rosa gallica* flower)

Xiangyang Guo

College of Tea and Food Science, Xinyang Normal University, Xinyang 464000, China;

xiangyang.guo@ahau.edu.cn

Reagents and materials

The fresh flowers of *Rosa gallica* L. were collected from Zhengzhou Botanical Garden, a natural growing area in Zhengzhou City, Henan Province, China. The sampling was conducted in the early morning before sunrise during the full flowering stage in May 2022, with healthy, pest- and disease-free plants with fully bloomed flowers selected as the sampling materials to ensure the quality of plant samples. The collected fresh flowers of *Rosa gallica* L. were formally authenticated as the genuine species by Prof. Hui Zhu, a botany expert from Hanshan Normal University. All collection activities of the plant materials strictly complied with the national and local relevant regulations on the protection and utilization of wild plants in China, and no special collecting permits were required for this plant species as it is not a protected plant under the Convention on the Trade in Endangered Species of Wild Fauna and Flora (CITES) or national key protected wild plant species in China.

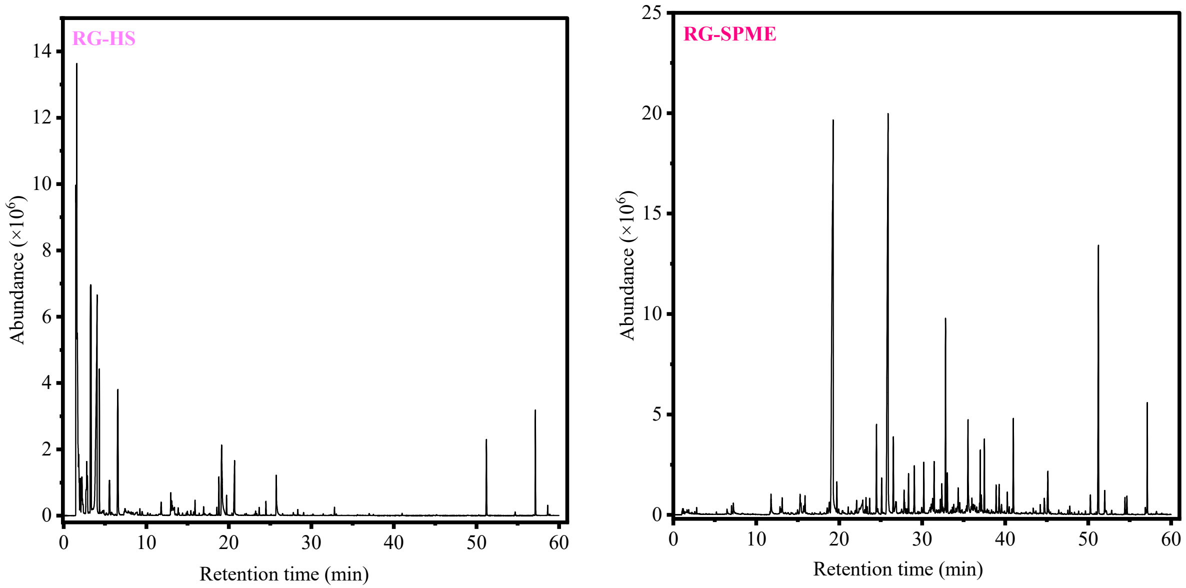

Figure S1 Representative total ion current chromatograms of RG-HS and RG-SPME.

Table S1 The related information of chemicals used in the research

Chemicals	Related information			
	Puriety	Supplier	City	Country
Dimethyl sulfide	≥99%	Sigma Aldrich	Shanghai	China
Pentanal	>97%	Sigma Aldrich	Shanghai	China
Hexanal	>98%	Sigma Aldrich	Shanghai	China
2-Hexenal	≥95%	Sigma Aldrich	Shanghai	China
1-Hexanol	≥99%	Sigma Aldrich	Shanghai	China
Heptanal	≥95%	Sigma Aldrich	Shanghai	China
(*E,E*)-2,4-Hexadienal	>95%	Sigma Aldrich	Shanghai	China
Benzaldehyde	≥99%	Sigma Aldrich	Shanghai	China
(E,E)-2,4-Heptadienal	≥90%	Sigma Aldrich	Shanghai	China
Benzyl alcohol	≥98%	Sigma Aldrich	Shanghai	China
cis-*β*-Ocimene	≥90%	Sigma Aldrich	Shanghai	China
γ-Terpinene	≥95%	Sigma Aldrich	Shanghai	China
Linalool	>95%	Sigma Aldrich	Shanghai	China
Nonanal	>95%	Sigma Aldrich	Shanghai	China
Phenylethyl alcohol	≥99%	Sigma Aldrich	Shanghai	China
(*E,Z*)-2,6-Nonadienal	≥96%	Sigma Aldrich	Shanghai	China
Methyl salicylate	≥98%	Sigma Aldrich	Shanghai	China
α-Terpineol	≥96%	Sigma Aldrich	Shanghai	China
Geraniol	≥98%	Sigma Aldrich	Shanghai	China
cis-Citral	≥96%	Sigma Aldrich	Shanghai	China
trans-Citral	≥96%	Sigma Aldrich	Shanghai	China
(*E*)-3,7-Dimethyl-2,6-octadienal	≥96%	Sigma Aldrich	Shanghai	China
Indole	≥99%	Sigma Aldrich	Shanghai	China
Eugenol	>99%	Sigma Aldrich	Shanghai	China
trans-Isoeugenol	>98%	Sigma Aldrich	Shanghai	China
trans-*β*-Ionone	≥97%	Sigma Aldrich	Shanghai	China
α-Farnesene	≥90%	Sigma Aldrich	Shanghai	China
Nerolidol	>98%	Sigma Aldrich	Shanghai	China
2,3-Butanedione	≥99%	Aladdin Chemicals	Shanghai	China
2-Methylfuran	≥98%	Aladdin Chemicals	Shanghai	China
3-Methylbutanal	≥99%	Aladdin Chemicals	Shanghai	China
2-Methylbutanal	≥95%	Aladdin Chemicals	Shanghai	China
Acetic acid	≥99%	Aladdin Chemicals	Shanghai	China
1-Pentanol	≥98%	Aladdin Chemicals	Shanghai	China
(*E*)-2-Hexenal	≥98%	Aladdin Chemicals	Shanghai	China
Styrene	≥99%	Aladdin Chemicals	Shanghai	China
2-Acetylfuran	≥99%	Aladdin Chemicals	Shanghai	China
α-Pinene	≥98%	Aladdin Chemicals	Shanghai	China
Camphene	≥95%	Aladdin Chemicals	Shanghai	China
6-Methyl-5-hepten-2-one	≥98%	Aladdin Chemicals	Shanghai	China
β-Pinene	≥97%	Aladdin Chemicals	Shanghai	China
Hexanoic acid	≥99%	Aladdin Chemicals	Shanghai	China
Octanal	≥97%	Aladdin Chemicals	Shanghai	China
α-Terpinene	≥90%	Aladdin Chemicals	Shanghai	China
Acetophenone	≥99%	Aladdin Chemicals	Shanghai	China
Ethyl octanoate	≥99%	Aladdin Chemicals	Shanghai	China
Decanal	≥97%	Aladdin Chemicals	Shanghai	China
Ethyl nonanoate	≥95%	Aladdin Chemicals	Shanghai	China
Ethyl decanoate	≥99%	Aladdin Chemicals	Shanghai	China
trans-Geranylacetone	≥97%	Aladdin Chemicals	Shanghai	China
Ethyl dodecanoate	≥99%	Aladdin Chemicals	Shanghai	China
Farnesol	≥95%	Aladdin Chemicals	Shanghai	China

Furfural	>99.5%	Aladdin Chemicals	Shanghai	China
2-Furanmethanol	>98%	Aladdin Chemicals	Shanghai	China
β-Myrcene	≥90%	Aladdin Chemicals	Shanghai	China
Limonene	>95%	Aladdin Chemicals	Shanghai	China
Benzeneacetaldehyde	>95%	Aladdin Chemicals	Shanghai	China
β-Ocimene	≥90%	Aladdin Chemicals	Shanghai	China
n-alkanes (C5-C28)	Analytical grade	Supelco	Bellefonte, PA	USA

Table S2 Odor quality, odor threshold value, and relative amounts of the identified volatiles in *Rosa gallica* flowers from different extraction methods

No.	Volatile compounds	RT (min)	RI-1	RI-2	Relative abundance (%)		Odor quality ψ	Threshold (mg/m^3) #	Odor type
					RG-HS	RG-SPME			
1	Dimethyl sulfide*	1.37	535	527	nd	0.0101±0.0007	Sulfury, foul-smelling, chestnut-like	0.3	Sulfury
2	Ethyl Acetate*	1.84	576	586	nd	0.1281±0.007	Fruity	0.88	Fruity
3	2-Methylpropanal	1.96	558	552	3.2602±0.0867	nd	Fresh, aldehydic, floral, green, herbal	0.001	Green
4	2,3-Butanedione*	2.11	597	595	3.2622±0.5167	nd	Butter-like	0.00018	Dairy
5	2-Methylfuran*	2.18	603	606	0.2159±0.0249	nd	Nutty, sweet, musty	200	Nutty
6	2-Butenal	2.59	641	642	nd	0.0627±0.0087	Pungent	0.067	Chemical
7	3-Methylbutanal*	2.71	652	652	0.9368±0.0908	nd	Malty, fruity, apple/peach-like	0.00035	Fruity
8	2-Methylbutanal*	2.79	659	662	4.6932±0.1161	nd	Pungent, coffee/cocoa-like, fruity	0.1	Fruity
9	2,3-Pentanedione	3.24	701	698	0.4201±0.0633	nd	Buttery, caramel-like, nutty, sweet, creamy, cheesy	0.02	Dairy
10	Pentanal*	3.26	701	699	18.5644±3.1209	0.1251±0.0074	Fermented, winey, bready, fruity, nutty, berry	0.85	Fruity
11	Acetic acid*	4.03	736	729	22.9001±1.1239	nd	Pungent, vinegar-like	0.013	Chemical
12	1-Hydroxy-2-propanone	4.32	748	738	2.1753±0.2377	nd	Pungent, sweet, caramel-like, green	10000	Chemical
13	1-Pentanol*	4.72	766	65	0.4983±0.08	nd	Fusel oil-like (faint)	0.153	Chemical
14	Hexanal*	5.54	801	804	2.3892±0.3105	0.0445±0.0073	Grassy, green, fresh, fatty	0.23	Green
15	Dihydro-2-methyl-3(2H)-furanone	5.79	808	809	0.318±0.0528	nd	Nutty, creamy, almond	23000	Nutty
16	Furfural*	6.55	829	833	9.5607±0.3703	0.1612±0.0036	Sweet, bready, caramel-like	2.8	Sweet
17	2-Hexenal*	7.03	842	835	nd	0.1521±0.0094	Grassy, herbal	0.48	Green
18	(*E*)-2-Hexenal*	7.24	848	854	nd	0.3093±0.0469	Green, leafy, fruity	0.0031	Green
19	2-Furanmethanol*	7.82	865	859	1.0911±0.0758	nd	Burnt, sweet, bready, caramel-like	32	Sweet
20	4-Cyclopentene-1,3-dione	8.37	880	881	0.3155±0.0162	nd	——	n.f.	——
21	Styrene*	8.65	887	893	nd	0.0089±0.0012	Floral	26.4	Floral
22	Pentanoic acid	8.88	894	904	0.386±0.019	0.0404±0.0072	Unpleasant flavor, smelly	0.00016	Chemical
23	Heptanal*	9.22	903	901	0.3108±0.0106	nd	Green, oily, grassy	0.26	Green
24	2-Acetylfuran*	9.50	909	911	0.2884±0.0446	nd	Nutty, sweet, roasted, baked	15025.2	Roasted
25	(*E,E*)-2,4-Hexadienal*	9.57	910	911	nd	0.0268±0.0007	Green, sweet, fruity, waxy, fatty	0.0018	Green
26	*α*-Pinene*	10.47	930	937	0.1247±0.0111	nd	Pine-like	0.1	Woody
27	Camphene*	11.19	946	952	0.0629±0.0076	nd	Woody, herbal, camphor-like, terpenic	30	Woody
28	5-Methyl-2-furanmethanol	11.53	953	958	0.1609±0.017	nd	Roasted, meaty, baked potato-like	32	Roasted

29	5-Methyl-2-furancarboxaldehyde	11.80	959	960	0.7597±0.0286	nd	Sweet, caramel-like, bready, brown, coffee-like	500	Roasted
30	Benzaldehyde*	11.80	959	962	0.1227±0.0218	0.6486±0.0176	Almond-like, fruity, cherry-like, powdery, nutty	0.085	Floral
31	6-Methyl-5-hepten-2-one*	12.97	985	986	0.9592±0.1288	0.269±0.0185	Fruity, apple-like, citrus	0.01889	Fruity
32	*β*-Pinene*	13.04	986	979	nd	0.0583±0.0085	Pine-like	0.18	Woody
33	*β*-Myrcene*	13.12	988	984	nd	0.3606±0.0693	Woody, resinous, musty, balsamic, ethereal	0.1125	Woody
34	Hexanoic acid*	13.39	994	990	1.0491±0.1451	nd	Acrid flavor	0.0048	Chemical
35	*trans*-2-(2-Pentenyl)furan	13.57	998	1003	0.0864±0.005	nd	Meaty	1000	Roasted
36	Decane*	13.67	1000	1000	0.0641±0.0031	nd	Alkane-like	4000000	Chemical
37	Octanal*	13.86	1004	1004	0.3603±0.0609	nd	Fatty, green, citrus, waxy	0.17	Fatty
38	*α*-Phellandrene	13.87	1004	1005	0.3603±0.0557	0.0308±0.0028	Spicy, pepper-like	3.9	Spicy
39	(*E*)-3-Hexen-1-ol acetate	13.96	1006	1008	0.1262±0.0245	nd	Fresh, green, fruity, sweet	870	Fruity
40	(*E,E*)-2,4-Heptadienal*	14.21	1011	1012	nd	0.0437±0.0022	Fatty, green, oily, cinnamon-like	0.057	Green
41	*α*-Terpinene*	14.39	1015	1010	0.1361±0.0143	nd	Citrusy, woody, lemon-like	7.9	Citrus
42	2-Pyrrolaldehyde	14.62	1020	1015	0.0419±0.0082	nd	Meaty, roasted, nutty	65000	Roasted
43	*o*-Cymene	14.77	1023	1022	0.0364±0.007	0.0436±0.0048	Aromatic	0.005	Aromatic
44	Limonene*	14.98	1027	1026	0.2194±0.0118	0.1579±0.0302	Citrus, lemon, orange, green, etherel	0.21	Citrus
45	*β*-Phellandrene	15.02	1028	1029	0.0058±0.0006	nd	Turpentine, terpenic, minty	36	Green
46	Benzyl alcohol*	15.28	1033	1036	nd	0.6264±0.1136	Fruity, rose-like	2546.21	Floral
47	*cis*-*β*-Ocimene*	15.40	1036	1035	0.2334±0.0204	0.3371±0.0588	Citrus, herbal, spicy, sweet	0.01	Citrus
48	Benzeneacetaldehyde*	15.74	1043	1038	0.1783±0.0061	0.2518±0.0353	Floral, rose, cherry-like	6.3	Floral
49	*β*-Ocimene*	15.90	1046	1037	0.5806±0.0174	0.5786±0.0457	Citrus, herbaceous, sweet	0.0187	Citrus
50	*γ*-Terpinene*	16.42	1056	1060	0.1157±0.0033	0.0291±0.0045	Citrus, lemon-like, woody, spicy, juicy	55.0	Citrus
51	Acetophenone*	16.74	1063	1065	nd	0.0432±0.001	Sweet, cherry pit, vanilla-like	1990	Sweet
52	Pineapple ketone	16.78	1064	1059	0.0038±0.0005	nd	Sweet, caramel-like, strawberry-like	0.002	Fruity
53	2-Acetylpyrrole	16.95	1067	1064	0.4097±0.0716	nd	Nutty, musty	> 2	Roasted
54	Heptanoic acid	17.62	1081	1078	0.1687±0.0023	nd	Rancid, sour, cheesy, sweat, fatty	0.022	Chemical
55	Terpinolene	17.72	1083	1088	0.1197±0.0024	0.0688±0.009	Fresh, woody, sweet, piney, citrus	200	Woody
56	2-Ethenyl-1,4-dimethylbenzene	17.97	1088	1095	nd	0.01±0.0014	Solvent-like, aromatic	0.25	Chemical
57	*p*-Cymenene	18.11	1091	1091	nd	0.0147±0.0004	Phenolic, spicy, styrene, eugenol and guaiacol-like with musty, coffee and nutty nuances	2.66	Spicy
58	Methyl benzoate	18.18	1092	1097	nd	0.0583±0.0091	Floral, fruity	0.0015	Floral
59	Linalool*	18.55	1100	1103	0.0687±0.011	0.1921±0.0138	Floral, sweet	0.0024	Floral

60	Nonanal*	18.80	1105	1104	1.4553±0.1837	0.4956±0.0085	Floral, green, lemon-like	0.0031	Floral
61	Phenylethyl Alcohol*	19.27	1115	1116	7.309±0.1862	25.558±0.8399	Floral, rose-like	0.021	Floral
62	Methyl octanoate	19.73	1125	1126	0.7947±0.1106	0.7407±0.0911	Waxy, green, sweet, vegetable-like, herbal	200	Green
63	Cosmene	19.93	1128	1132	nd	0.204±0.0209	Herb, citrus	n.f.	Green
64	(*E,Z*)-2,6-Dimethyl-2,4,6-octatriene	20.42	1139	1131	nd	0.1246±0.0134	Sweet, floral, nutty, herbal, peppery	0.02	Floral
65	Pyranone	20.98	1150	1151	0.0512±0.0028	nd	Hay-like	n.f.	Woody
66	(*E,Z*)-2,6-Nonadienal*	21.08	1152	1153	nd	0.1713±0.0292	Green, fatty, dry cucumber-like, melon-like	0.00011	Green
67	(*E*)-2-Nonenal	21.44	1160	1157	nd	0.0716±0.0102	Cucumber, green	0.00009	Green
68	Phenylethyl formate	22.10	1174	1178	0.1459±0.0046	0.3632±0.0044	Green, rose-like, hyacinth/watercress-like, herbal	270	Floral
69	Octanoic acid	22.83	1189	1180	nd	1.1025±0.0926	Fatty, waxy, rancid, oily, cheesy, vegetable-like	0.0051	Fatty
70	Methyl salicylate*	22.86	1189	1192	nd	0.0175±0.0029	Peppermint, wintergreen mint	0.016	Green
71	*α*-Terpineol*	23.09	1194	1189	0.0698±0.002	0.1139±0.0217	Pleasant, floral	0.86	Floral
72	Ethyl octanoate*	23.24	1197	1194	0.1859±0.0096	0.3451±0.0342	Fruity, pineapple-like, sweet	0.04	Fruity
73	Dodecane*	23.38	1200	1200	0.094±0.0099	0.1662±0.0074	Alkane-like	0.77	Chemical
74	Decanal*	23.67	1206	1205	0.3354±0.0106	0.3447±0.0478	Sweet, citrus, waxy, floral	0.0026	Sweet
75	*α*-4-Dimethyl-3-cyclohexene-1-acetaldehyde	24.13	1216	1217	0.0421±0.0059	nd	——	n.f.	——
76	Benzothiazole	24.35	1221	1229	nd	0.0207±0.0036	Meaty, vegetative, brown, beefy, coffee-like	80	Roasted
77	Methyl nonanoate	24.49	1224	1225	0.5519±0.0719	1.8067±0.103	Sweet, fruity, pear-like, waxy, winey	0.048	Fruity
78	Citronellol	24.64	1227	1229	nd	0.1811±0.0233	Fresh, rose-like	0.0465	Floral
79	*cis*-Citral*	25.11	1237	1241	nd	0.7017±0.1213	Citrus, lemon-like	0.00015	Citrus
80	Dimethyl hexanedioate	25.38	1243	1243	nd	0.0539±0.0083	Nutty	50	Nutty
81	3,7-Dimethyl-2,6-octadien-1-ol	25.89	1254	1260	nd	17.9772±0.6277	Fresh, floral, rose-like, citrus, sweet	0.6	Floral
82	Phenylethyl acetate	25.97	1256	1258	0.0196±0.0024	0.0066±0.001	Floral, rosy, with a slight balsamic nuance	0.0002	Floral
83	Geraniol*	26.10	1259	1263	2.5191±0.214	0.0958±0.0066	Rose-like, sweet, honey-like	0.6	Floral
84	*trans*-Citral*	26.53	1268	1270	0.0927±0.0112	1.5989±0.1957	Citrus, lemon-like	0.00015	Citrus
85	Nonanoic acid	26.81	1274	1266	nd	0.8496±0.0676	Fatty (faint), coconut-like	0.12	Fatty
86	Anethole	27.31	1285	1286	nd	0.0504±0.0076	Star anise-like, sweet	0.057	Spicy
87	Indole*	27.52	1289	1289	nd	0.2167±0.0143	Floral, animal-like	0.0081	Floral
88	Ethyl nonanoate*	27.82	1296	1296	0.1899±0.0254	0.6904±0.0623	Fruity, rose-like, waxy, soapy, winey	0.01	Fruity
89	Tridecane*	28.00	1300	1300	0.0432±0.0069	0.1367±0.0233	Alkane-like	42	Chemical
90	Undecanal	28.35	1308	1309	0.2371±0.0067	0.8021±0.0465	Waxy, soapy, floral, aldehydic, citrus, green, fatty	0.14	Waxy

91	2,6,10,10-Tetramethyl-1-oxaspiro[4.5]dec-6-ene	28.51	1311	1302	0.0444±0.0053	nd	Woody, cooling, minty, herbal	n.f.	Woody
92	Methyl decanoate	29.06	1324	1325	0.1199±0.0091	1.0715±0.0908	Fatty, oily, fruity	8.8	Fatty
93	*α*-Cubebene	29.94	1344	1345	nd	0.2531±0.0375	Spicy, citrus	n.f.	Spicy
94	Eugenol*	30.20	1350	1357	0.0789±0.0028	1.1096±0.1492	Sweet, spicy, clove-like, woody	0.00061	Floral
95	Isoledene	30.96	1367	1373	nd	0.2571±0.0099	Pine-like, spicy	n.f.	Woody
96	3-Methyltridecane	31.09	1370	1370	nd	0.2226±0.0118	Alkane-like	> 42	Chemical
97	*α*-Copaene	31.17	1372	1376	nd	0.1813±0.0334	Spicy	0.1	Spicy
98	1-Undecanol	31.26	1374	1374	nd	0.423±0.0469	Fresh, waxy, rose-like, soapy, floral	46	Floral
99	*cis*-Geranyl acetate	31.44	1378	1367	0.0866±0.0006	1.1106±0.0173	Neroli-like, rose-like, honey, raspberry-like	18	Floral
100	2,4-Diisopropenyl-1-methyl-1-vinylcyclohexane	31.79	1386	1398	nd	0.0971±0.0094	Star anise-like, spicy	n.f.	Spicy
101	1-Tetradecene	32.04	1392	1397	nd	0.0885±0.0136	——	60	——
102	Ethyl decanoate*	32.19	1395	1396	nd	0.3071±0.0546	Sweet, waxy, fruity, apple/grape-like, oily, brandy	0.53	Fruity
103	Methyleugenol	32.38	1400	1402	0.0584±0.0071	0.9573±0.1588	Clove-like, anise-like, carnation-like	8500	Floral
104	Tetradecane*	32.39	1400	1400	nd	0.2117±0.0115	Alkane-like	5	Chemical
105	Cyperene	32.50	1402	1399	nd	0.1899±0.0087	Spicy	n.f.	Spicy
106	1,3,5-Trimethoxybenzene	32.68	1407	1418	nd	0.1242±0.0182	Aromatic	1.5	Aromatic
107	Dodecanal	32.80	1410	1409	0.3298±0.0641	4.2835±0.3365	Soapy, waxy, citrus	0.033	Waxy
108	Caryophyllene	33.03	1415	1419	0.0822±0.0133	0.9848±0.1865	Woody, green, spicy, terpenic	13	Woody
109	Methyl undecanoate	33.38	1423	1429	nd	0.0805±0.0117	Fatty, waxy, fruity	0.0003	Fatty
110	*β*-copaene	33.47	1426	1426	nd	0.1106±0.007	Spicy	0.1	Spicy
111	*β*-Guaiene	33.81	1434	1447	nd	0.2939±0.0551	Earthy, spicy, woody	n.f.	Woody
112	Aromandendrene	33.97	1438	1440	nd	0.045±0.0046	Citrus, orange-like	n.f.	Citrus
113	*α*-Elemene	34.13	1442	1454	nd	0.1181±0.0106	Herbal, waxy, fresh	n.f.	Green
114	*trans*-Isoeugenol*	34.25	1444	1454	nd	0.172±0.0125	Clove-like	6	Floral
115	*trans*-Geranylacetone*	34.33	1446	1452	nd	0.6403±0.1066	Fresh, rose-like, floral, green, fruity	60	Floral
116	Humulene	34.53	1451	1457	nd	0.3338±0.0281	Woody	160	Woody
117	*γ*-Muurolene	34.71	1455	1472	nd	0.1026±0.0094	Floral, fragrance, Ylang-like	n.f.	Floral
118	(+)-epi-Bicyclosesquiphellandrene	34.80	1458	1445	nd	0.1257±0.011	Spicy, minty, herbal, woody	n.f.	Spicy
119	*γ*-Selinene	35.27	1469	1479	nd	0.168±0.0177	Celery-like, green, spicy	1	Green
120	1-Dodecanol	35.52	1475	1478	nd	2.4177±0.3159	Earthy, soapy, waxy, fatty, honey, coconut-like	1.2	Waxy

121	*trans*-*β*-Ionone*	35.54	1476	1478	nd	0.0103±0.0003	Violet, raspberry, floral	0.007	Floral
122	*D*-Germacrene	35.59	1477	1482	0.0655±0.0091	0.252±0.0215	Woody, spicy	15	Woody
123	*β*-Selinene	35.90	1484	1482	nd	0.2394±0.0034	Celery-like	1	Green
124	Ledene	35.99	1486	1489	nd	0.3814±0.0438	Pine-like, spicy	n.f.	Woody
125	*δ*-Cadinene	36.09	1489	1498	nd	0.1312±0.024	Herbal, woody	0.222	Green
126	*α*-Selinene	36.19	1491	1491	nd	0.2762±0.0124	Celery-like	1	Green
127	*α*-Muurolene	36.33	1494	1499	nd	0.3712±0.0568	Ylang-like, fragrance	0.002	Floral
128	Pentadecane*	36.55	1500	1500	nd	0.189±0.0318	Alkane	> 13000000	Chemical
129	*α*-Farnesene*	36.67	1503	1508	nd	0.073±0.0012	Woody, green, floral, herbal	87	Floral
130	2,5-Bis(1,1-dimethylethyl)phenol	36.76	1505	1514	nd	0.1005±0.0028	Aromatic	n.f.	Aromatic
131	2-Isopropyl-5-methyl-9-methylenebicyclo[4.4.0]dec-1-ene	36.91	1509	1510	nd	0.2122±0.0066	——	n.f.	——
132	Tridecanal	37.00	1511	1512	0.0871±0.0095	1.2687±0.0247	Fresh, aldehydic, soapy, waxy, grapefruit peel	10	Waxy
133	*β*-Cadinene	37.13	1514	1518	0.0235±0.0035	0.5007±0.0712	Minty, camphoraceous, herbaceous, woody, phenolic, warm	n.f.	Green
134	*cis*-Calamenene	37.26	1518	1531	nd	0.1097±0.0073	Citrus (faint), green	0.05	Citrus
135	Methyl dodecanoate	37.51	1524	1526	0.0527±0.0034	1.5142±0.1007	Waxy, soapy, creamy, coconut-like	0.0026	Waxy
136	1,2,3,4,4a,7-Hexahydro-1,6-dimethyl-4-(1-methylethyl)naphthalene	37.69	1529	1533	nd	0.1591±0.0018	——	2	——
137	*α*-Cadinene	37.84	1532	1538	nd	0.186±0.0148	Minty, camphoraceous, herbaceous, woody, phenolic, warm	n.f.	Green
138	*α*-Calacorene	38.02	1537	1542	nd	0.1086±0.004	Woody	n.f.	Woody
139	Elemicine	38.36	1546	1554	nd	0.1544±0.0076	Spicy	n.f.	Spicy
140	Nerolidol*	38.91	1560	1556	nd	0.6681±0.0754	Floral, green, citrus, woody, waxy	10000	Floral
141	*cis*-3-Hexenyl benzoate	39.27	1569	1565	nd	0.7654±0.0075	Green, leafy, floral, balsamic, fatty	5000	Green
142	*n*-Tridecan-1-ol	39.56	1576	1577	nd	0.3311±0.0089	Solvent-like	> 42000	Chemical
143	Ethyl dodecanoate*	40.26	1594	1593	nd	0.5722±0.1067	Sweet, waxy, soapy, rummy with a creamy, floral nuance	0.002	Sweet
144	Hexadecane*	40.50	1600	1600	nd	0.1896±0.0114	Alkane-like	0.5	Chemical
145	Lauryl acetate	40.82	1608	1609	nd	0.147±0.0215	Sweet, oily, waxy, soapy, creamy	0.41	Sweet
146	Tetradecanal	40.99	1613	1613	0.1059±0.0072	1.9497±0.0837	Fatty, waxy, dry citrus peel-like, musky	110	Fatty
147	Methyl tridecanoate	41.39	1624	1624	nd	0.0538±0.0052	Waxy, soapy, fatty	> 5700	Waxy
148	*tau*-Muurolol	42.00	1640	1642	nd	0.0333±0.0052	Ylang-like, fragrance, floral	200000	Floral
149	*α*-Cadinol	42.42	1651	1653	nd	0.0416±0.0068	Woody, herbal	200000	Woody

150	(*E,E*)-3,7,11-Trimethyl-2,6-dodecadien-1-ol	42.53	1654	1661	nd	0.0768±0.0154	Floral, fresh, sweet	n.f.	Floral
151	*Ar*-Tumerone	42.76	1660	1664	nd	0.0292±0.0037	——	n.f.	——
152	1-Tetradecanol	43.39	1677	1685	nd	0.2448±0.0464	Fruity, waxy, coconut-like	> 5000	Fruity
153	Heptadecane*	44.25	1700	1700	0.0242±0.0044	0.221±0.0221	Alkane-like	10000000	Chemical
154	Farnesol*	44.72	1713	1713	nd	0.5139±0.0169	Floral, fresh, sweet	1000	Floral
155	*trans*-Farnesal	45.46	1734	1737	nd	0.0792±0.0075	Floral, minty	82	Floral
156	Benzyl Benzoate	46.46	1762	1762	nd	0.1323±0.0166	Floral, sweet, balsamic	0.0006	Floral
157	Guiazulene	46.64	1767	1775	nd	0.0145±0.0024	Chamomile-like	n.f.	Citrus
158	1-Octadecene	46.88	1774	1788	nd	0.0339±0.0033	——	n.f.	——
159	Ethyl tetradecanoate	47.56	1793	1794	nd	0.1013±0.0079	Fatty, waxy	0.5	Waxy
160	Octadecane*	47.80	1800	1800	nd	0.1762±0.027	Alkane-like	0.02	Chemical
161	Hexadecanal	48.35	1816	1815	nd	0.053±0.0022	Cardboard-like	n.f.	Woody
162	Methyl pentadecanoate	48.63	1824	1820	nd	0.0133±0.0011	Waxy, fatty	> 500	Waxy
163	(*E*)-Farnesyl acetate	48.85	1831	1834	nd	0.0906±0.0049	Green, floral, orchid-like, waxy, rose-like, citrus	n.f.	Floral
164	Phenylethyl octanoate	49.23	1842	1851	nd	0.0476±0.0017	Sweet, waxy, green, fruity, fruity winey	10000	Sweet
165	1-Nonadecene	50.75	1887	1883	nd	0.027±0.0035	——	n.f.	——
166	9-Nonadecene	50.96	1893	1893	0.0369±0.0019	0.065±0.008	——	n.f.	——
167	Nonadecane*	51.21	1900	1900	2.9456±0.3567	6.4374±0.4381	Alkane-like	10000000	Chemical
168	Methyl hexadecanoate	52.00	1923	1926	nd	0.5085±0.0455	Oily, waxy, fatty	> 2000	Waxy
169	Ethyl hexadecanoate	54.44	1993	1994	nd	0.3779±0.0261	Oily, waxy, fatty	2000	Waxy
170	Eicosane	54.69	2000	2000	0.1593±0.0148	0.3859±0.055	Alkane-like	10000000	Chemical
171	10-Heneicosene (c,t)	56.90	2091	2107	0.0382±0.001	nd	——	n.f.	——
172	Methyl linolenate	57.03	2096	2098	nd	0.0826±0.0123	Fatty, waxy	450	Waxy
173	Heneicosane	57.14	2100	2100	2.9195±0.3681	1.5718±0.2338	Alkane-like	10000000	Chemical
174	Methyl stearate	57.60	2127	2126	nd	0.0224±0.0004	Waxy, oily	> 500	Waxy
175	Ethyl linoleate	58.14	2158	2163	nd	0.0181±0.0003	Oily, fatty, woody	450	Waxy
176	Ethyl linolenate	58.25	2164	2165	nd	0.0695±0.0026	Fatty, waxy	450	Waxy
177	Butyl hexadecanoate	58.63	2186	2188	0.3714±0.0197	nd	Waxy	> 2000	Waxy
178	Ethyl octadecanoate	58.75	2193	2197	nd	0.0088±0.0009	Waxy	> 500	Waxy
179	Docosane*	58.88	2200	2200	0.059±0.0029	0.0225±0.0016	Alkane-like, waxy	10000000	Chemical
180	1-Tricosene	60.60	2289	2291	nd	0.0071±0.001	——	n.f.	——

181	Tricosane*	60.82	2300	2300	nd	0.1045±0.0194	Alkane-like	10000000	Chemical

RI-1, the retention index (RI) was computed by using *n*-alkanes (C5-C28) under the same chromatographic conditions with the detected volatile compounds; RI-2, the data was from the literature (http://webbook.nist.gov/chemistry/).
The compounds indicated with '*' were identified using authentic standard compounds.
ψ Odor description found in the literature (Flavornet; The LRI and Odour Database), and no human sensory evaluation with panelists was conducted in this work.
'——', no odor description information was found in the literature.
All the odor thresholds were obtained from: 'Odour & Flavour Detection Thresholds in Water (In Parts per Billion, l μg/L)' (http://www.leffingwell.com/odourthre.htm); Ho et al., 2015; Guo, Ho, Wan, Zhu, Liu, & Wen, 2021a; Guo, Ho, Schwab, & Wan, 2021b; Guo, Ho, Schwab, & Wan, 2021c; Guo, Schwab, Ho, Song, & Wan, 2022.
'n.f.', data was not found in the literature.
nd, not detectable.

Table S3 The common volatiles identified in *Rosa gallica* flowers from different extraction methods

No.	Volatile compounds	RT (min)	RI	Relative abundance (%)	
				RG-HS	RG-SPME
1	Pentanal	3.26	701	18.5644±3.1209	0.1251±0.0074
2	Hexanal	5.54	801	2.3892±0.3105	0.0445±0.0073
3	Furfural	6.55	829	9.5607±0.3703	0.1612±0.0036
4	Pentanoic acid	8.88	894	0.386±0.019	0.0404±0.0072
5	Benzaldehyde	11.80	959	0.1227±0.0218	0.6486±0.0176
6	6-Methyl-5-hepten-2-one	12.97	985	0.9592±0.1288	0.269±0.0185
7	*α*-Phellandrene	13.87	1004	0.3603±0.0557	0.0308±0.0028
8	*o*-Cymene	14.77	1023	0.0364±0.007	0.0436±0.0048
9	Limonene	14.98	1027	0.2194±0.0118	0.1579±0.0302
10	*cis*-*β*-Ocimene	15.40	1036	0.2334±0.0204	0.3371±0.0588
11	Benzeneacetaldehyde	15.74	1043	0.1783±0.0061	0.2518±0.0353
12	*β*-Ocimene	15.90	1046	0.5806±0.0174	0.5786±0.0457
13	*γ*-Terpinene	16.42	1056	0.1157±0.0033	0.0291±0.0045
14	Terpinolene	17.72	1083	0.1197±0.0024	0.0688±0.009
15	Linolool	18.55	1100	0.0687±0.011	0.1921±0.0138
16	Nonanal	18.80	1105	1.4553±0.1837	0.4956±0.0085
17	Phenylethyl Alcohol	19.27	1115	7.309±0.1862	25.558±0.8399
18	Methyl octanoate	19.73	1125	0.7947±0.1106	0.7407±0.0911
19	Phenylethyl formate	22.10	1174	0.1459±0.0046	0.3632±0.0044
20	*α*-Terpineol	23.09	1194	0.0698±0.002	0.1139±0.0217
21	Ethyl octanoate	23.24	1197	0.1859±0.0096	0.3451±0.0342
22	Dodecane	23.38	1200	0.094±0.0099	0.1662±0.0074
23	Decanal	23.67	1206	0.3354±0.0106	0.3447±0.0478
24	Methyl nonanoate	24.49	1224	0.5519±0.0719	1.8067±0.103
25	Phenylethyl acetate	25.97	1256	0.0196±0.0024	0.0066±0.001
26	Geraniol	26.10	1259	2.5191±0.214	0.0958±0.0066
27	*trans*-Citral	26.53	1268	0.0927±0.0112	1.5989±0.1957
28	Ethyl nonanoate	27.82	1296	0.1899±0.0254	0.6904±0.0623
29	Tridecane	28.00	1300	0.0432±0.0069	0.1367±0.0233
30	Undecanal	28.35	1308	0.2371±0.0067	0.8021±0.0465
31	Methyl decanoate	29.06	1324	0.1199±0.0091	1.0715±0.0908
32	Eugenol	30.20	1350	0.0789±0.0028	1.1096±0.1492
33	*cis*-Geranyl acetate	31.44	1378	0.0866±0.0006	1.1106±0.0173
34	Methyleugenol	32.38	1400	0.0584±0.0071	0.9573±0.1588
35	Dodecanal	32.80	1410	0.3298±0.0641	4.2835±0.3365
36	Caryophyllene	33.03	1415	0.0822±0.0133	0.9848±0.1865
37	*D*-Germacrene	35.59	1477	0.0655±0.0091	0.252±0.0215
38	Tridecanal	37.00	1511	0.0871±0.0095	1.2687±0.0247
39	*β*-Cadinene	37.13	1514	0.0235±0.0035	0.5007±0.0712
40	Methyl dodecanoate	37.51	1524	0.0527±0.0034	1.5142±0.1007
41	Tetradecanal	40.99	1613	0.1059±0.0072	1.9497±0.0837
42	Heptadecane	44.25	1700	0.0242±0.0044	0.221±0.0221
43	9-Nonadecene	50.96	1893	0.0369±0.0019	0.065±0.008
44	Nonadecane	51.21	1900	2.9456±0.3567	6.4374±0.4381
45	Eicosane	54.69	2000	0.1593±0.0148	0.3859±0.055
46	Heneicosane	57.14	2100	2.9195±0.3681	1.5718±0.2338
47	Docosane	58.88	2200	0.059±0.0029	0.0225±0.0016

Table S4 The rOAV values of identified volatiles in *Rosa gallica* flowers from different extraction methods

No.	Volatile compounds	rOAV	
		RG-HS	RG-SPME
1	Dimethyl sulfide	0.00	0.00
2	Ethyl Acetate	0.00	0.00
3	2-Methylpropanal	17.99	0.00
4	2,3-Butanedione	100.00	0.00
5	2-Methylfuran	0.00	0.00
6	2-Butenal	0.00	0.01
7	3-Methylbutanal	14.77	0.00
8	2-Methylbutanal	0.26	0.00
9	2,3-Pentanedione	0.12	0.00
10	Pentanal	0.12	0.00
11	Acetic acid	9.72	0.00
12	1-Hydroxy-2-propanone	0.00	0.00
13	1-Pentanol	0.02	0.00
14	Hexanal	0.06	0.00
15	Dihydro-2-methyl-3(2H)-furanone	0.00	0.00
16	Furfural	0.02	0.00
17	2-Hexenal	0.00	0.00
18	(*E*)-2-Hexenal	0.00	0.94
19	2-Furanmethanol	0.00	0.00
20	4-Cyclopentene-1,3-dione	nd	nd
21	Styrene	0.00	0.00
22	Pentanoic acid	13.31	2.37
23	Heptanal	0.01	0.00
24	2-Acetylfuran	0.00	0.00
25	(*E,E*)-2,4-Hexadienal	0.00	0.14
26	(*R*)-*α*-Pinene	0.01	0.00
27	Camphene	0.00	0.00
28	5-Methyl-2-furanmethanol	0.00	0.00
29	5-Methyl-2-furancarboxaldehyde	0.00	0.00
30	Benzaldehyde	0.01	0.07
31	6-Methyl-5-hepten-2-one	0.28	0.13
32	*β*-Pinene	0.00	0.00
33	*β*-Myrcene	0.00	0.03
34	Hexanoic acid	1.21	0.00
35	*trans*-2-(2-Pentenyl)furan	0.00	0.00
36	Decane	0.00	0.00
37	Octanal	0.01	0.00
38	*α*-Phellandrene	0.00	0.00
39	(*E*)-3-Hexen-1-ol acetate	0.00	0.00
40	(*E,E*)-2,4-Heptadienal	0.00	0.01
41	*α*-Terpinene	0.00	0.00
42	2-Pyrrolaldehyde	0.00	0.00
43	*o*-Cymene	0.04	0.08
44	Limonene	0.01	0.01
45	*β*-Phellandrene	0.00	0.00
46	Benzyl alcohol	0.00	0.00
47	*cis*-*β*-Ocimene	0.13	0.32
48	Benzeneacetaldehyde	0.00	0.00
49	*β*-Ocimene	0.17	0.29
50	*γ*-Terpinene	0.00	0.00

51	Acetophenone	0.00	0.00
52	Pineapple ketone	0.01	0.00
53	2-Acetylpyrrole	0.00	0.00
54	Heptanoic acid	0.04	0.00
55	Terpinolene	0.00	0.00
56	2-Ethenyl-1,4-dimethylbenzene	0.00	0.00
57	*p*-Cymenene	0.00	0.00
58	Methyl benzoate	0.00	0.36
59	Linalool	0.16	0.75
60	Nonanal	2.59	1.50
61	Phenylethyl Alcohol	1.92	11.42
62	Methyl octanoate	0.00	0.00
63	Cosmene	nd	nd
64	(*E,Z*)-2,6-Dimethyl-2,4,6-octatriene	0.00	0.06
65	Pyranone	nd	nd
66	(*E,Z*)-2,6-Nonadienal	0.00	14.61
67	(*E*)-2-Nonenal	0.00	7.47
68	Phenylethyl formate	0.00	0.00
69	Octanoic acid	0.00	2.03
70	Methyl salicylate	0.00	0.01
71	*α*-Terpineol	0.00	0.00
72	Ethyl octanoate	0.03	0.08
73	Dodecane	0.00	0.00
74	Decanal	0.71	1.24
75	*α*-4-Dimethyl-3-cyclohexene-1-acetaldehyde	nd	nd
76	Benzothiazole	0.00	0.00
77	Methyl nonanoate	0.06	0.35
78	Citronellol	0.00	0.04
79	*cis*-Citral	0.00	43.89
80	Dimethyl hexanedioate	0.00	0.00
81	3,7-Dimethyl-2,6-octadien-1-ol	0.00	0.28
82	Phenylethyl acetate	0.54	0.31
83	Geraniol	0.02	0.00
84	*trans*-Citral	3.41	100.00
85	Nonanoic acid	0.00	0.07
86	Anethole	0.00	0.01
87	Indole	0.00	0.25
88	Ethyl nonanoate	0.10	0.65
89	Tridecane	0.00	0.00
90	Undecanal	0.01	0.05
91	2,6,10,10-Tetramethyl-1-oxaspiro[4.5]dec-6-ene	nd	nd
92	Methyl decanoate	0.00	0.00
93	*α*-Cubebene	nd	nd
94	Eugenol	0.71	17.07
95	Isoledene	nd	nd
96	3-Methyltridecane	0.00	0.00
97	*α*-Copaene	0.00	0.02
98	1-Undecanol	0.00	0.00
99	*cis*-Geranyl acetate	0.00	0.00
100	2,4-Diisopropenyl-1-methyl-1-vinylcyclohexane	nd	nd
101	1-Tetradecene	0.00	0.00
102	Ethyl decanoate	0.00	0.01
103	Methyleugenol	0.00	0.00

104	Tetradecane	0.00	0.00
105	Cyperene	nd	nd
106	1,3,5-Trimethoxybenzene	0.00	0.00
107	Dodecanal	0.06	1.22
108	Caryophyllene	0.00	0.00
109	Methyl undecanoate	0.00	2.52
110	*β*-copaene	0.00	0.01
111	*β*-Guaiene	nd	nd
112	Aromandendrene	nd	nd
113	*α*-Elemene	nd	nd
114	*trans*-Isoeugenol	0.00	0.00
115	*trans*-Geranylacetone	0.00	0.00
116	Humulene	0.00	0.00
117	*γ*-Muurolene	nd	nd
118	(+)-epi-Bicyclosesquiphellandrene	nd	nd
119	*γ*-Selinene	0.00	0.00
120	1-Dodecanol	0.00	0.02
121	*trans*-*β*-Ionone	0.00	0.01
122	*D*-Germacrene	0.00	0.00
123	*β*-Selinene	0.00	0.00
124	Ledene	nd	nd
125	*δ*-Cadinene	0.00	0.01
126	*α*-Selinene	0.00	0.00
127	*α*-Muurolene	0.00	1.74
128	Pentadecane	0.00	0.00
129	*α*-Farnesene	0.00	0.00
130	2,5-Bis(1,1-dimethylethyl)phenol	nd	nd
131	2-Isopropyl-5-methyl-9-methylenebicyclo[4.4.0]dec-1-ene	nd	nd
132	Tridecanal	0.00	0.00
133	*β*-Cadinene	nd	nd
134	*cis*-Calamenene	0.00	0.02
135	Methyl dodecanoate	0.11	5.46
136	1,2,3,4,4a,7-Hexahydro-1,6-dimethyl-4-(1-methylethyl)naphthalene	0.00	0.00
137	*α*-Cadinene	nd	nd
138	*α*-Calacorene	nd	nd
139	Elemicine	nd	nd
140	Nerolidol	0.00	0.00
141	*cis*-3-Hexenyl benzoate	0.00	0.00
142	*n*-Tridecan-1-ol	0.00	0.00
143	Ethyl dodecanoate	0.00	2.68
144	Hexadecane	0.00	0.00
145	Lauryl acetate	0.00	0.00
146	Tetradecanal	0.00	0.00
147	Methyl tridecanoate	0.00	0.00
148	*tau*-Muurolol	0.00	0.00
149	*α*-Cadinol	0.00	0.00
150	(*E,E*)-3,7,11-Trimethyl-2,6-dodecadien-1-ol	nd	nd
151	*Ar*-Tumerone	nd	nd
152	1-Tetradecanol	0.00	0.00
153	Heptadecane	0.00	0.00
154	Farnesol	0.00	0.00
155	*trans*-Farnesal	0.00	0.00

156	Benzyl Benzoate	0.00	2.07
157	Guiazulene	nd	nd
158	1-Octadecene	nd	nd
159	Ethyl tetradecanoate	0.00	0.00
160	Octadecane	0.00	0.08
161	Hexadecanal	nd	nd
162	Methyl pentadecanoate	0.00	0.00
163	(*E*)-Farnesyl acetate	nd	nd
164	Phenylethyl octanoate	0.00	0.00
165	1-Nonadecene	nd	nd
166	9-Nonadecene	nd	nd
167	Nonadecane	0.00	0.00
168	Methyl hexadecanoate	0.00	0.00
169	Ethyl hexadecanoate	0.00	0.00
170	Eicosane	0.00	0.00
171	10-Heneicosene (c,t)	nd	nd
172	Methyl linolenate	0.00	0.00
173	Heneicosane	0.00	0.00
174	Methyl stearate	0.00	0.00
175	Ethyl linoleate	0.00	0.00
176	Ethyl linolenate	0.00	0.00
177	Butyl hexadecanoate	0.00	0.00
178	Ethyl octadecanoate	0.00	0.00
179	Docosane	0.00	0.00
180	1-Tricosene	nd	nd
181	Tricosane	0.00	0.00

nd, not detectable.
